# Presence of HER4 associates with increased sensitivity to Herceptin™ in patients with metastatic breast cancer

**DOI:** 10.1186/bcr2339

**Published:** 2009-07-22

**Authors:** Andrea Sassen, Simone Diermeier-Daucher, Manuela Sieben, Olaf Ortmann, Ferdinand Hofstaedter, Stephan Schwarz, Gero Brockhoff

**Affiliations:** 1 Institute of Pathology, University of Regensburg, Franz-Josef-Strauss-Allee 11, 93053 Regensburg, Germany; 2 Department of Gynecology and Obstetrics, University of Regensburg, Franz-Josef-Strauß-Allee 11, D-93053 Regensburg, Germany; 3 Institute of Pathology, University of Erlangen, Krankenhausstr. 12, 91054 Erlangen, Germany

## Abstract

**Introduction:**

HER2 overexpression, or rather *HER2 *gene amplification, is indicative for Herceptin therapy in both metastatic and pre-metastatic breast cancer patients. Patient's individual sensitivity to Herceptin treatment, however, varies enormously and spans from effectual responsiveness over acquired insensitivity to complete resistance from the outset. Thus no predictive information can be deduced from HER2 determination so that molecular biomarkers indicative for Herceptin sensitivity or resistance need to be identified. Both ErbB receptor-dependent signalling molecules as well as HER2-related ErbB receptor tyrosine kinases, known to mutually interact and to cross-regulate each other are prime candidates to be involved in cellular susceptibility to Herceptin.

**Methods:**

Using immunohistochemistry and fluorescence *in situ *hybridisation, we retrospectively investigated primary breast cancer tissues from 48 patients who were under Herceptin treatment. We quantified the gene copy numbers of all *HER *receptors and evaluated their coexpression profile. Moreover the HER2 phosphorylation state, the ratio of native to truncated HER2, p27(kip1) and PTEN expression were objects of this study.

**Results:**

Above all markers investigated in this study Kaplan-Meier and Cox regression analysis revealed a significant positive impact of HER4 (co-)expression on overall survival from beginning of antibody therapy. Both HER4 expression and *HER4 *gene amplification emerged as independent prognostic markers in Herceptin-treated breast cancer patients and responsiveness to Herceptin turned out to be more efficient if tumour cells show HER4 expression.

**Conclusions:**

Although HER4 is known to potentially exert a tumour cell killing activity and in turn to have a favourable impact in breast cancer patients we demonstrate here the first time that HER4 expression prolongs overall survival in Herceptin-treated patients. Elucidating HER4 receptor function in the context of Herceptin treatment will advance the design of highly efficient receptor targeting. By then we need to extend the analysis of breast cancer by allowing for HER2/HER4 coexpression by which valuable additional prognostic and predictive information might possibly be revealed.

## Introduction

The overexpressed human epidermal growth factor receptor (HER) 2 receptor tyrosine kinase (RTK) is a useful therapeutic target in breast cancer patients. In pathological diagnostics, the HER2 expression level is routinely evaluated and an elevated receptor expression (scored 3+) is usually based on *HER2 *gene amplification, which can reliably be identified by *in situ *hybridisation [1]. Without contraindication HER2 protein overexpression (or *HER2 *gene amplification) represents the worldwide accepted rationale for antibody-targeted therapy using trastuzumab (Herceptin™). Herceptin™ was approved in Europe in 2000 for the treatment of breast cancer patients suffering from metastatic and aggressive tumour growth that is indicative for a poor disease outcome [2]. In fall 2006, Herceptin™ treatment was extended by the approval for adjuvant patient treatment [3]. Statistically considered, antigen-specific targeting of tumour cells has brought significant clinical benefit to these patients, both in terms of overall survival (OS) and recurrence free survival (RFS) documented in many clinical trials [4,5]. Although HER2 overexpression or *HER2 *gene amplification is a prerequisite for therapeutic antibody treatment, the extent of sensitivity to Herceptin™ therapy varies enormously and ranges from excellent individual responsiveness over acquired insensitivity to complete resistance from the outset [6-9]. Consequently, HER2 overexpression does not represent a reliable predictive marker for responsiveness to Herceptin™.

Numerous molecular biomarkers implicated in individual sensitivity to Herceptin™ treatment have been suggested, but have not been precisely and reproducibly defined to date. The panel of suggested molecules comprises proteins involved in intracellular survival signalling (PTEN) [10] and cell proliferation (p21, p27) [11-15], as well as cell membrane located molecules featuring the capacity to directly interact with HER2 and thereby modulate lateral and transversal HER2 activity [16]. Among them the HER2-related RTK family members HER1 (also known as epidermal growth factor receptor (EGFR)), HER3 and HER4 are in the focus of interest [17]. Depending on their coexpression profile HER receptors act in concert on ligand binding and trigger transmembraneous signal transduction as a functional unit rather than individually and independently. The recruitment of intracellular signalling pathways is a result of intensively cross-talking receptors and well-defined three-dimensional intracellular activation domains [18]. Hence a highly organised spatiotemporal coexpression of HER receptors plays a pivotal role in growth, development and differentiation both on the cellular and organ level in healthy organisms [19,20]. However, HER1 and/or HER2 overexpression, as well as atypical HER receptor coexpression profiles, causing receptor hyperactivation and enhanced mitogenic signalling, could have been frequently attributed to malignant cell growth [17].

Moreover, HER3 has been found to preferentially interact with HER2 [21] and, although kinase deficient, to significantly enhance mitogenic signalling [22]. As a consequence even antiproliferative effects mediated by Herceptin™ can be extenuated by HER3 coexpression [23]. Remarkably, receptor coexpression does not necessarily result in hyperstimulation and hypermitogenic cellular activity. Signal modulation, fine tuning and control can also be achieved by extensive receptor communication and even antiproliferative and induction of differentiation responses on growth factor binding (i.e. heregulin) to HER4 have been previously described [24,25].

Conceivably the tissue-specific coexpression of HER receptors has immediate impact on the cellular response to Herceptin™, which specifically binds to HER2. In a previous study we have experimentally shown that the cellular sensitivity to Herceptin™ treatment is directly contingent on the EGFR coexpression density and can be enhanced by EGFR downregulation [26]. In another study undertaken in our laboratory, we have shown that amplified *HER3 *and *HER4 *gene copy numbers have additional prognostic impact on the course of breast cancer disease in patients both with and without *HER2 *gene amplification [27].

Here, in a series of 48 breast cancer patients with *HER2 *gene amplification we retrospectively investigated numerous additional biomolecular parameters to have potential impact on the course of disease under Herceptin™ treatment (prognostic value) and to be implicated in responsiveness to Herceptin™ treatment (predictive value). Therefore, we quantified the gene copy numbers of all *HER *receptors via fluorescent *in situ *hybridisation (FISH) and immunohistochemically investigated the coexpression profile of all HER receptors. In addition, the HER2 phosphorylation state, the ratio of native to truncated HER2, the expression of p27^kip1 ^and PTEN, and the presence of the proliferation associated antigen Ki-67 were studied. As inhibition of activated HER2 might be primarily responsible for Herceptin™ sensitivity rather than the total receptor targeting [28], we evaluated the pY1248 phosphorylation, which has been linked to the mitogenic ras/MAP kinase signalling pathway [29]. Herceptin™ is targeted to the juxtamembrane domain of HER2 [30], so the loss of binding site by receptor truncation could be suggestive for antibody resistance [31]. Furthermore, the identification of proteolytically shed HER2 (sHER2/p95) in relation to total HER2 expression could be informative in terms of receptor activation and consequently the efficacy of Herceptin™ binding. Moreover PTEN, a negative regulator of PKB/Akt, and p27^kip1 ^both downstream targets of HER2, have been proposed to be involved in Herceptin™ resistance [10,32].

The aim of this study was to cytogenetically and immunohistochemically identify molecular biomarkers within the context of HER receptor signalling, which affects the susceptibility of metastatic breast cancer to Herceptin™ treatment. Of all the markers investigated in this study, the presence of truncated and phosphorylated receptors appeared to have an impact by trend on the course of disease of Herceptin™-treated (HT) patients. All Kaplan-Meier and Cox regression analyses revealed a significant positive impact of HER4 (co-)expression on OS from the beginning of antibody therapy. Finally both HER4 expression and *HER4 *gene amplification emerged as independent prognostic markers in HT breast cancer patients. HER4 receptor expression is even supposed to provide predictive value because it apparently affects susceptibility to Herceptin™ treatment.

## Materials and methods

This study was approved by the Institutional Review Board of the University of Regensburg, Germany.

### Breast tumour samples and patient characteristics

Formalin-fixed paraffin-embedded tissue blocks from 48 female patients with invasive lobular or ductal unilateral primary breast cancer (median age, 56.0 years; range 31.4 to 80.9) were obtained from the archives of the Institute of Pathology, Regensburg, and the Institute of Pathology, Hospital Weiden, Germany. All patients were diagnosed between March 1997 and October 2005 and underwent Herceptin™ therapy between September 2000 and November 2005. The patients were not involved in any clinical trial. Clinical data were acquired by the Tumour Centre Inc., Regensburg. The median follow-up period was 55.8 months (range 28.4 to 116.6). The median OS time was 41.7 months (range 10.6 to 116.6), median RFS was 21.9 months (range 1.5 to 63.6). A total of 36 patients (75%) died and 12 patients survived until the end of monitoring. Of the 48 patients, 31 (65%) suffered from a recurrence of breast cancer including the subgroups of 4 of the 12 surviving patients (33.3%) plus 27 of the 36 (75%) patients who passed away.

### Fluorescence *in situ *hybridisation

Tissue microarrays (TMAs) were constructed and handled as described previously [27]. Briefly, after dewaxing and rehydrating, slides were steamed in sodium citrate. Cell structures were digested in pepsin and hydrochloric acid, then washed and dehydrated.

For FISH analyses, 5 μm sections of the TMAs directly labelled dual-colour DNA probes for *HER1 *(7p11.2), *HER2 *(17q21-22), *HER3 *(12q13.2), *HER4 *(2q33.3-34), and *oestrogen receptor α *(*ERα*, 6q25) (ZytoVision Ltd., Bremerhaven, Germany) were used. The probes identified locus-specific sequences for both the genes and the corresponding centromeres 7, 17, 12, 2, and 6 to differentiate between gene amplification and polysomy of the respective chromosome. Respective DNA probe sets were applied to the TMA area and incubated overnight at 37°C. Subsequent to several washing steps, nuclei were counterstained with DAPI (4',6-diamidino-2-phenylindole) and analysed by epifluorescence microscopy. FISH scoring was performed by counting fluorescence signals in 25 malignant, non-overlapping cell nuclei for each case by two independent interpreters (AS, MB). The FISH ratio was assessed as the number of genes proportional to the number of centromeres. Patients with *HER2 *ratios, that is total gene/total centromere relations, of 1.5 or more were considered as amplified based on recent statistical approaches of our group [27] and included in this study.

TMA sections stained with H&E were used for reference histology.

### Microscopy, fluorescence *in situ *hybridisation scoring and digital imaging

Slides were imaged as described previously [27] with an Axio Imager Z.1 (Zeiss, Göttingen, Germany), equipped with specific filter sets (AHF, Tübingen, Germany) and the plug-in module ApoTome™ for taking pseudoconfocal images. Three-dimensional *z*-stacks were generated, colours were separately recorded and digitally processed. Corresponding images were superimposed.

### Immunohistochemistry

Immunostaining with anti-HER-receptor antibodies, anti-phosphoHER2, anti-extracellular HER2, anti-ER, anti-progesterone receptor (PR), anti-p27^kip1^, and anti-PTEN was performed on 5 μm sections of the TMAs and applied in accordance with the manufacturer's instructions. Table 1 explicitly shows the antibody-specific staining and scoring characteristics. Interpretation was performed independently by two experienced pathologists (SS, FH). Stably transfected mouse fibroblasts proved specific immunostaining of HER1 to HER4 [27].

**Table 1:** Characteristics for immunostaining and scoring

Protein	HER2 EC	phospho-HER2 (pY^1248^)	p27^Kip1^	Oestrogen receptor α	Progesterone receptor
Antibody	Rabbit mAb	Mouse mAb	Mouse mAb	Mouse mAb	Mouse mAb
Origin	Thermo Scientific	Thermo Scientific	Lab Vision	Novocastra	Novocastra
Clone	SP3	PN2A, Ab-18	DCS-72.F6, Ab-1	6F11	1A6
Dilution of primary antibody	1:50	1:100	1:100	1:60	1:40
Staining pattern	Membrane		Nucleus and cytoplasm	Nucleus	
Epitope retrieval	Heat induced, 10 mM citric acid buffer, pH 7.3		Heat induced, 10 mM sodium-citric acid pH 6.0	Heat induced, 10 mM citric acid buffer, pH 7.3	
Blocking	Endogenous peroxidase blocking				
Primary antibody	30 min, room temperature	Overnight, 4°C	30 min, room temperature	60 min, room temperature	
Detection system	EnVision™ Dual Link System; DAB + chromogenic substrate			*i*VIEW™ DAB Detection Kit	
Scoring in accordance with	HercepTest guidelines		Immunoreactive score	Manufacturer's guidelines	

Anti-HER1 to 4 and anti-Ki-67 antibodies were applied as described previously [27].

EC = antibody binds to extracellular domain; HER = human epidermal growth factor receptor; mAb = monoclonal antibody.

Anti HER1 to 4 and anti-Ki67 antibodies were assessed as described previously [27]. HER2 phosphorylation status was scored as suggested in the Herceptest™ guidelines. The classification into p27^Kip1 ^positive (n = 24) and negative cases (n = 24) was performed using an immunoreactive score (IRS), defined by the following formula: nuclear staining (0 to 100%) × intensity of staining (0 = no staining, 1 = weak staining, 2 = moderate staining, 3 = strong staining) plus percentage of cells with cytoplasmic staining × medial staining intensity. An IRS of at least 200 was regarded as positive. Anti-PTEN staining (Rabbit monoclonal antibody, clone 138G6, Cell Signaling, Boston, MA, USA) detected endogeneous levels of total protein analogously to the above mentioned procedure considering nuclear and cytoplasmic staining, respectively. ER and PR scoring was performed according to Remmele and Stegner [33]. Anything but IRS 0 was considered positive.

### Statistical analyses

The primary outcome measure, OS, was calculated as the time from the beginning of Herceptin™ treatment to death from any cause or to the date on which the patient was last known to be alive. Patients lost to follow-up were treated as censored cases on the basis of the last contact date. A secondary outcome measure, RFS, was omitted because in our series Herceptin™ treatment was primarily indicated after diagnosis of relapse and/or in patients with metastatic breast cancer (M1, R2) so that complete remission did not occur.

Survival curves were generated using the Kaplan-Meier method, and log-rank tests compared the distributions between groups. In addition, hazard ratios (HR) with 95% confidence intervals (CI) were estimated for covariates (treated as continuous, and where appropriate as a dichotomous variable) using the Cox proportional-hazards model. Non-parametric correlations were analysed by Spearman tests. In all analyses, *P *≤ 0.05 (two-tailed) was considered significant. Statistical analyses were performed with SPSS version 16.0 (SPSS Inc., Chicago, IL, USA).

## Results

### Data validation

**Figure 1:**
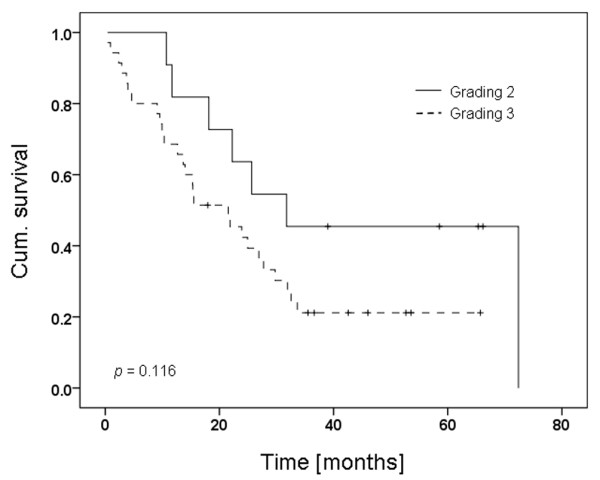
Kaplan-Meier curve of tumour grading in Herceptin™-treated breast cancer patients. *P *= 0.116.

TMAs of paraffin-embedded samples from 48 HT breast cancer patients were used to analyse gene amplification and protein expression of each member of the HER family. Metastasis as a prerequisite for patients receiving Herceptin™ therapy is referred to as stage IV (defined as Tx, Nx, M1). We found one grade 1 tumour (not included in the statistics), but 11 graded 2 (according to [34]) and 35 graded 3 with a trend for longer survival with tumours graded 2 (*P *= 0.116, Figure 1). For OS, patients age at various dichotomisations (cut-off points at 45, 50, 55 and 60 years) played no significant role (< 50 years, n = 20 vs. ≥ 50 years, n = 28; *P *= 0.399).

Univariate Cox analysis (Table 2) revealed HER4 (FISH and immunohistochemistry analysed), phosphoHER2 and p27^Kip1 ^(immunohistochemistry) all to have a positive prognostic impact on OS. A multivariate Cox analysis (backstep: LR) was accomplished enclosing all parameters including *HER1, 3, 4, and ERα*, FISH, HER1, 3, 4 immunohistochemistry, Ki67 immunohistochemistry, ER and PR immunohistochemistry, grading, and age. The final step is shown in Table 3.

**Table 2:** Univariate Cox proportional hazards analysis

	Parameter	Overall survival		
		HR	95% CI	*P*
FISH	*HER1*	0.45	0.12 to 1.787	0.256
	*HER3*	1.14	0.34 to 37.51	0.943
	***HER4***	<0.01	<0.01 to 0.33	**0.011**
	*ERα*	1.19	0.71 to 2.00	0.511
IHC	HER1	1.62	0.67 to 3.92	0.282
	HER2 shedding	1.36	0.69 to 2.70	0.376
	**phospho-HER2**	0.20	0.05 to 0.86	**0.030**
	HER3	1.01	0.42 to 2.44	0.982
	**HER4**	0.43	0.22 to 0.85	**0.016**
	Ki-67	1.36	0.67 to 2.77	0.397
	ER	1.00	0.92 to 1.08	0.979
	PR	0.98	0.91 to 1.06	0.647
	**p27**^Kip1^	0.42	0.21 to 0.84	**0.014**
Others	Grading	2.01	0.83 to 4.87	0.123
	Age (<50 vs. ≥ 50 years)	1.34	0.68 to 2.67	0.401

Overall survival was defined from beginning of Herceptin™ treatment. Hazard ratios (HR), confidence intervals (CI) and *P *values of investigated parameters dependent on overall survival. CI with lower and upper limits. Data in bold define significant results.

ER = oestrogen receptor; FISH = fluorescence *in situ *hybridisation; HER = human epidermal growth factor receptor; IHC = immunohistochemistry.

**Table 3:** Last step of multivariate Cox proportional hazards analysis

Parameter	Overall survival		
	HR	95% CI	*P*
HER4 IHC	0.38	0.18 to 0.82	0.013
*HER4 *FISH	<0.01	<0.01 to 0.17	0.005
Grading	3.2	1.16 to 8.81	0.024

Overall survival was defined from beginning of Herceptin™ treatment, Cox analysis was performed stepwise backwards.

CI = confidence interval; HR = hazard ratio; FISH = fluorescence *in situ *hybridisation; HER = human epidermal growth factor receptor; IHC = immunohistochemistry.

### Fluorescence *in situ *hybridisation

*HER1*, *HER3*, *HER4*, and *ERα *were processed as continuous variables instead of dichotomising the data due to indefinable cut-off points [27]. *HER1, HER3*, and *ERα *FISH provided no further information whereas *HER4 *gene amplification was a significant positive marker for OS under Herceptin™ treatment (*HER4 *continuous: HR = 0.003 (95% CI < 0.01 to 0.17), *P *= 0.005, Table 3) in multivariate cox analysis.

### Immunohistochemistry of HER1, HER3, and HER4

All immunohistochemical results are shown in Table 4. Immunostaining of all four HER receptors was performed. The specificity of applied antibodies was proved by staining stably transfected mouse fibroblasts (NIH 3T3, kindly provided by Roche Diagnostics, Penzberg, Germany, data shown elsewhere [27]).

**Table 4:** Results of immunohistochemical stainings

Scoring	HER1 (n = 48)	HER3 (n = 47)	HER4 (n = 48)	phospho-HER2 (n = 43)	p27^Kip1 ^(n = 48)	ERα (n = 45)	PR (n = 46)
0	37 (77.1)	8 (16.7)	22 (45.8)	36 (83.7)	n/a	n/a	n/a
1	**7 (14.6)**	**31 (64.6)**	**7 (14.6)**	**3 (7.0)**	n/a	n/a	n/a
2	**3 (6.2)**	**8 (16.7)**	**12 (25.0)**	**4 (9.3)**	n/a	n/a	n/a
3	**1 (2.1)**	**0**	**7 (14.6)**	**0**	n/a	n/a	n/a
Negative	37 (77.1)	8 (16.7)	22 (45.8)	36 (83.7)	24 (50.0)	19 (42.2)	29 (63.0)
Positive	11 (22.9)	39 (81.2)	26 (54.2)	7 (16.3)	24 (50.0)	26 (57.8)	17 (37.0)

All 48 patients were *HER2 *FISH positive (ratio > 1.5). Numbers in parentheses are percentages. Results in bold define data considered positive for immunohistochemistry. n/a = not applicable, scoring defined in methodical chapter.

ER = oestrogen receptor; HER = human epidermal growth factor receptor; PR = Progesterone receptor.

By immunohistochemistry, 22.9% (11 of 48) were identified as HER1 positive (score 1+, 2+ and 3+) and 77.1% (37 of 48) as negative. In 22.4% (48 of 214) HER2 was overexpressed (score 2+ and 3+) and 77.6% (166 of 214) were unaltered. Immunohistochemistry of HER3 resulted in 93% (39 of 47) positive (score 1+, 2+ and 3+) and 17% (8 of 47) negative patients. Of the total number of cases, 54.2% (26 of 48) expressed HER4 (score 1+, 2+ and 3+), whereas 45.8% (22 of 48) did not.

**Figure 2:**
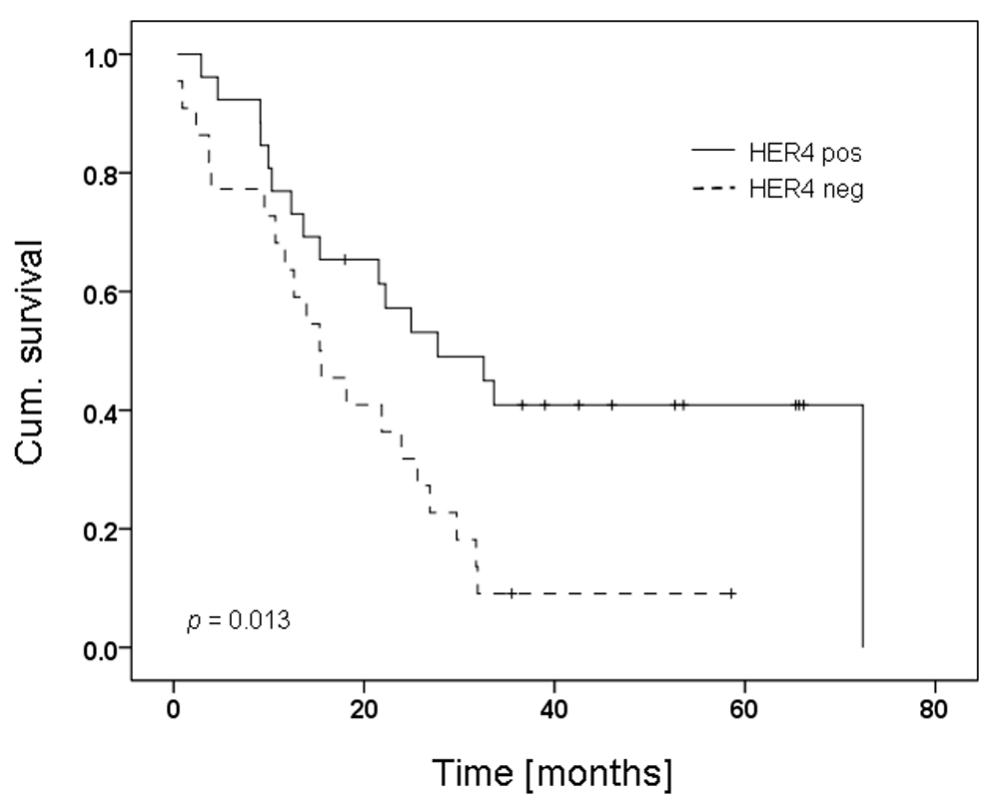
Kaplan-Meier curve of dichotomised HER4 IHC in Herceptin™-treated breast cancer patients. *P *= 0.013. HER4 negative = score 0; HER4 positive = score 1 to 3. HER = human epidermal growth factor receptor; IHC = immunohistochemistry.

As in the FISH results, HER1 and HER3 immunohistochemistry did not give further information (HER1 *P *= 0.278, HER3 *P *= 0.982; Kaplan-Meier curves not shown). Remarkably, HER4 immunohistochemistry corroborates *HER4 *FISH findings. HER4 expression resulted in significant longer survival times of HT patients (*P *= 0.013; Figure 2).

Within the patient collective investigated in this study, besides *HER4 *gene amplification and grading, HER4 immunohistochemistry was found to have significant positive impact on OS under Herceptin™ treatment (HR = 0.383 (95% CI 0.180 to 0.815), *P *= 0.013).

To evaluate Herceptin™ therapy, we matched the HT series (n = 48) with a cluster of patients (n = 20) originating from a recent study with no antibody-treated patients [27] and performed Kaplan-Meier survival analyses. The criterion for patient inclusion was their metastatic status (stage IV, M1 tumours).

**Figure 3:**
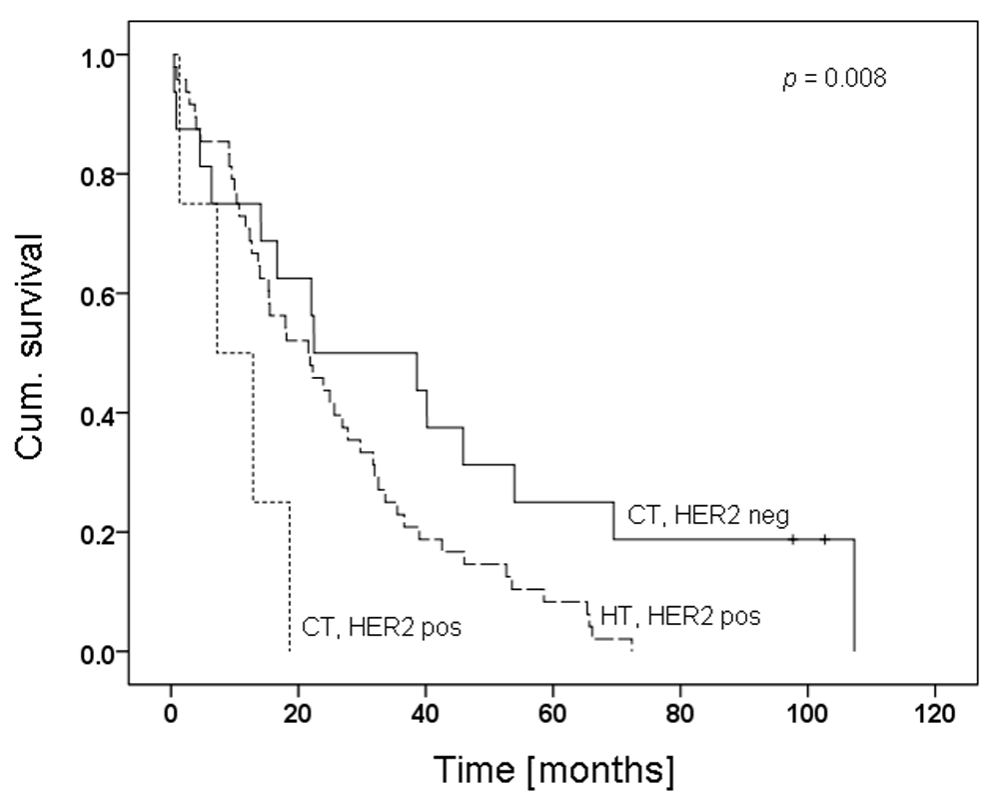
Kaplan-Meier curve of matched patient series. *P *= 0.008. Herceptin™ therapy (n = 48) compared with conventionally treated HER2 positive (n = 4) and conventionally treated HER2 negative (n = 16) patients. CT = conventional therapy; HER = human epidermal growth factor receptor; HT = Herceptin™ therapy.

Conventionally treated (CT) HER2-positive patients have the shortest survival time. Herceptin™ therapy improves their OS, but CT HER2-negative patients show the longest survival times (Figure 3). Irrespective of CT HER2-positive patients, a significant difference remains between HT HER2-positive patients and CT HER2-negative patients (*P *= 0.037). Thus, our statistical analysis is in agreement with the frequently found observation that HER2 positivity is a negative prognostic factor independent of therapy.

**Figure 4:**
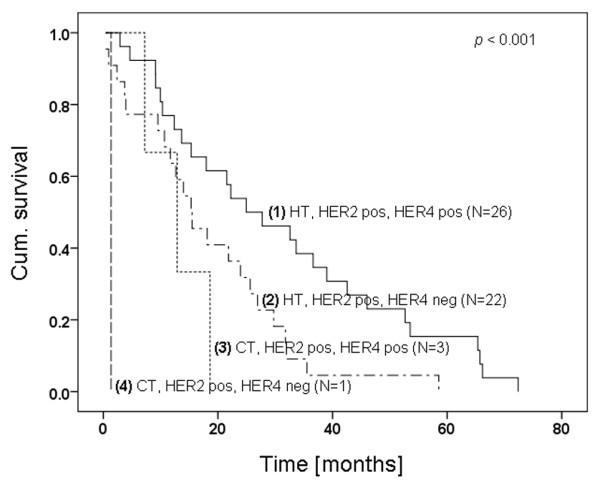
Kaplan-Meier curve of matched patient series with HER2 overexpression. *P *< 0.001. Herceptin™ therapy compared with conventionally treated HER2-negative patients as well as HER4 normal and overexpression. CT = conventional therapy; HER = human epidermal growth factor receptor; HT = Herceptin™ therapy.

Considering therapy options (CT and HT), Herceptin™ clearly ameliorated OS (Figure 4).

With given HER2 overexpression but independent of treatment, HER4 positivity lengthened survival times. HER4 positivity showed a positive impact on OS both in CT and in HT patients (curve 3 to 4, log rank, *P *= 0.013; and curve 1 to 2, log rank, *P *= 0.083).

Comparing patients with HT and HER4 negativity and patients with CT but HER4 positivity, no significant difference could be found (log rank, *P *= 0.351).

### Other immunohistochemical investigations

**Figure 5:**
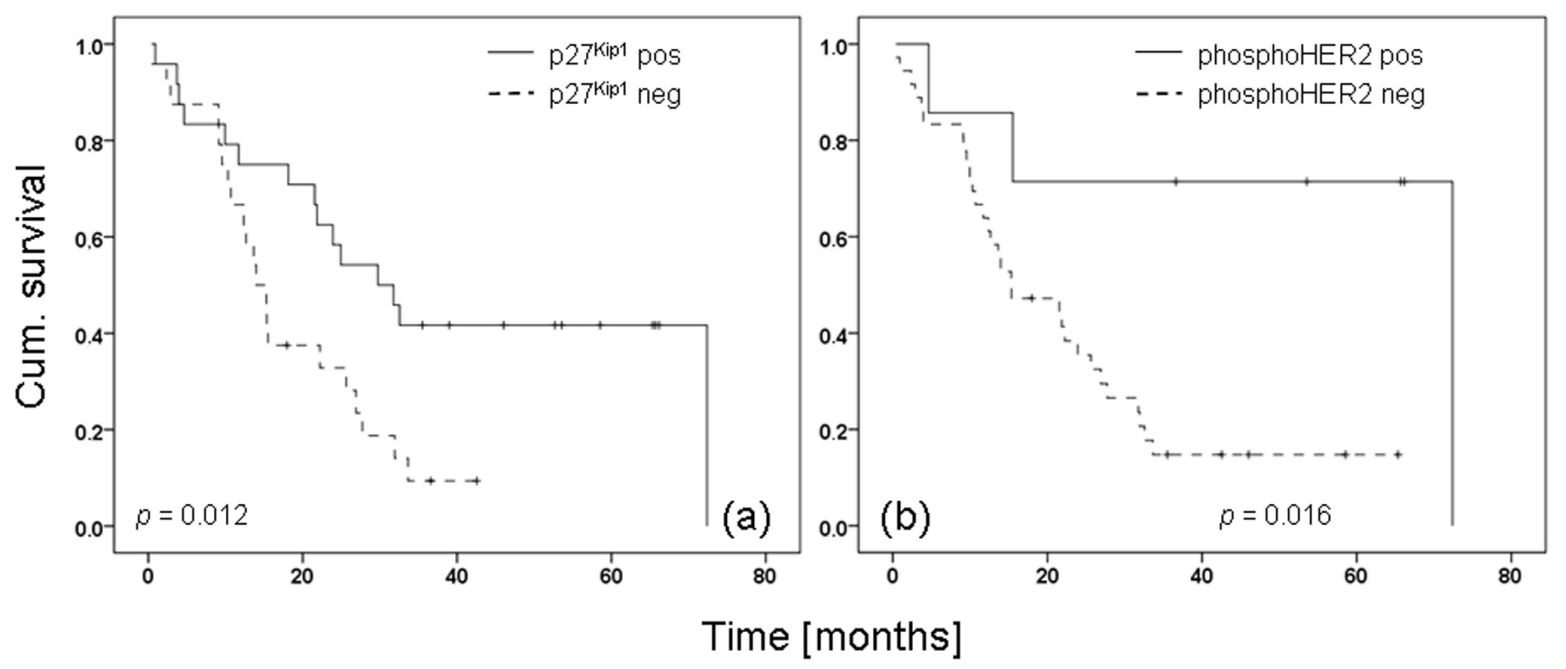
Kaplan-Meier curve of p27^Kip1 ^and HER2^pY1248 ^in Herceptin™-treated breast cancer patients. **(a) **Dichotomised p27^Kip1 ^immunohistochemistry (*P *= 0.012) and **(b) **phosphorylated HER2 receptor immunohistochemistry (*P *= 0.016). HER = human epidermal growth factor receptor.

Regarding the HT patient series, p27^Kip1 ^immunohistochemistry positive patients (n = 24) had a significantly better outcome concerning OS compared with p27^Kip1 ^negative (n = 24) patients (Figure 5a, log rank, *P *= 0.012).

Phosphorylated HER2 receptor (Figure 5b) was associated with increased OS (*P *= 0.016). In 16% of cases (7 of 43) we documented a positive staining with a significant positive effect on OS, whereas 84% of cases (36 of 43) showed less or no staining.

To examine the proliferation status in our patient cohort we assessed Ki-67 immunohistochemistry. The proliferative index had no significant impact on the OS of patients (data not shown).

All tumours were stained with two anti-HER2 antibodies. The routinely used antibody binds an intracellular target and showed a positive staining as expected in a HT patients series. We compared the staining intensity and pattern with staining obtained by using the SP3 antibody which targets a protruding extracellular HER2 domain (Table 4). In 22 cases we found differences; in 20 cases the extracellular staining was weaker than the intracellular, pointing out a shedding of the extracellular receptor domain. Comparing survival times of these 20 patients with those with homogeneous staining (n = 24), the latter demonstrated a trend for decreased RFS times (*P *= 0.096).

Immunohistochemical staining of ER and PR did not yield any supplemental information (ER *P *= 0.631, PR *P *= 0.593). The anti-PTEN antibody did not reveal any interpretable results and was eliminated from evaluation.

HER2 and ER immunohistochemistry were negatively correlated (*P *= 0.011, correlation coefficient = -0.374).

## Discussion

Screening of HER2 aberration (both on a protein or DNA level) as a prerequisite for Herceptin™ therapy does not allow the courses of disease or individual therapy responses to be predicted, so numerous biomarkers with potential impact on Herceptin™ responsiveness have moved into the focus of interest. Utilising immunohistochemistry and FISH, we retrospectively investigated the most likely parameters with immediate impact on HER2 activity and Herceptin™ responsiveness in a cohort of 48 HT breast cancer patients with HER2 amplification. Here, we present first-hand data comprising expression of all HER receptors as well as p27^Kip1 ^receptor phosphorylation and native vs. truncated HER2 staining.

Our study demonstrates for the first time that HER4 expression improves the outcome in a sub-collective of HER2-positive, HT breast cancer patients. First evidence indicating that, irrespective of therapy, HER4 coexpression has a positive impact on the outcome of breast cancer patients was published in 2003 [35], indicating a potential anti-tumourigenic effect mediated by the fourth member of the HER receptor family discovered in 1993 [25]. From there on a significant reduction of the proliferation indices in HER4-positive invasive breast carcinomas was reported, suggesting HER4 itself has a functional anti-proliferative or even protective capacity [36]. An association of HER4 expression and a favourable outcome in breast cancer patients has independently been described by Suo and colleagues [37] who found that HER4 coexpression obviously antagonises the effect of HER2 (over-)expression on the course of breast cancer disease.

Our observation that HER4 coexpression apparently favours the outcome of HER2-positive, HT breast cancer patients indicates that HER4 coexpression favours the HER2 targeting with Herceptin™. This suggests a non-autonomous role of HER4 but rather some degree of HER4/HER2 interplay what is in agreement with data provided by Sartor and colleagues [38]. They transfected HER2-positive cancer cells to overexpress HER4, achieving a reduction in proliferation and an increase in apoptosis which is suggestive of HER4 slowing down HER2 signalling activity. Moreover, a differentiation inducing effect by heregulin treatment in HER4 expressing SUM44 and SUM102 breast cancer cells has been experimentally demonstrated. This observation appeared to be independent of HER2 expression, because HER2 elimination could not eradicate the HER4 dependent decrease in cell growth. However, we previously found the coexpressed EGFR to contribute to cell susceptibility to Herceptin™ (and pertuzumab) and thereby provided evidence for EGFR/HER2 interaction playing a pivotal role in anti-HER2 targeting [26] in which HER4 is also most likely to be involved in.

Although HER4 expression has been frequently attributed to low-grade tumours with low proliferation activity [39], the discussion on the impact of HER4 on breast cancer progression and outcome of disease appeared contradictory over the past couple of years. This debate can be attributed to the ambivalent function of HER4 representing either oncogenic or tumour-suppressing activity [40]. Four differentially spliced HER4 isoforms have been identified but never been distinguished in descriptive studies addressing HER4 expression in primary cancer [41]. The JM-a/CYT-1 and JM-a/CYT-2 isoforms can be proteolytically cleaved by tumour necrosis factor-α converting enzyme [40,42] and subsequently by γ-secretase [43], whereas JM-b/CYT-1 and JM-b/CYT-2 variants represent non-cleavable counterparts [44] characterised by ligand-independent activity that promotes cancer cell growth [43]. Commonly, only JM-a/CYT-1 and JM-a/CYT-2 variants are expressed in tumour tissues at the same time [45]. The intracellular CYT isoforms differ by having (CYT-1) or not having (CYT-2) a cytoplasmic binding site for phosphoinositide 3-kinase. Upon cleavage of JM-a isoforms these intracellular released HER4 domains (4ICD) can be differentially translocated into intracellular compartments and, if cytoplasmically located, subsequently trigger anti-proliferative and pro-apoptotic signals [46,47]. In contrast, if translocated into the nucleus, they have the capacity to trigger pro-proliferative and survival promoting signalling [48-51] and accordingly a nuclear localisation of 4ICD has been associated to shortened survival of breast cancer patients [45]. In contrast, a mitochondrial accumulation of 4ICD, which evidently shares structural and functional homology with BH3-only pro-apoptotic BCL-2 family members, has also been reported [46]. This in turn results in a disturbance of mitochondrial membrane integrity. This process typically entails an apoptotic cell death characterised by mitochondrial permeabilisation, cytochrome-c efflux and caspase activation, and can explain the protective role of HER4.

The positive impact of HER4 on a patient's course of disease as shown in this study is not only suggestive of its differentiation promoting and potentially pro-apoptotic function but rather for a Herceptin™ augmenting effect. As HER2 is primarily phosphorylated on Herceptin™ treatment, as we have previously shown [52,16], it might be conceivable that HER4 can be crossactivated and a subsequent release of 4ICD might possibly enhance Herceptin™/HER2 triggered signalling. HER4, if coexpressed to HER2, could synergistically extenuate proliferative and survival signalling, for example via downregulation of ERK1/2 and PKB/Akt activity, respectively [53]. Numerous observations suggest that irrespective of any therapeutic treatment HER4 expression is associated with reduced tumour breast cancer aggressiveness, which is indicative of its prognostic impact [27,35-39,51,54]. Moreover, based on Kaplan-Meier analysis as illustrated in Figure 4, we here provide evidence that the outcome of HER2/HER4 double-positive patients is significantly better both under CT and HT compared with those patients who were HER2-positive but HER4 negative. The latter observation does not necessarily designate a predictive value of HER4 but is indicative of the positive effect of HER4 among patients characterised by a well defined therapeutic setting (either CT or HT treatment). Nevertheless, the molecular signalling background responsible for the higher efficiency of Herceptin™ treatment in HER2-positive/HER4-positive patients versus HER2-positive/HER4-negative patients needs to be experimentally elucidated in detail and the experimental design should allow for investigating the predictive relevance of HER4 in consideration of its oncogenic and tumour suppressing potential, respectively. Deciphering the molecular effect of HER4, which enhances therapy efficiency of Herceptin™ treatment, would affect the decision to undergo Herceptin™-related therapy but the presence of HER4 could be potentially therapeutically addressed in the future as well. It would not be advisable, for instance, to use novel anti-HER2 drugs, which would eradicate the therapy-enhancing effect of HER4. Alternatively one could consider therapeutically eliciting or intensifying the Herceptin™ enhancing effect of HER4.

The induction of the cyclin-dependent kinase inhibitor p27^Kip1 ^protein is considered as a key mechanism on anti-HER2 targeting using Herceptin™ by which at least six signalling targets and pathways might be modulated [12]. Also, in this study HER2 overexpressing patients with upregulated p27^Kip1^, benefit significantly from antibody therapy indicating a prognostic value of this protein in HT patients, which is in agreement with other reports [55,56].

Patients respond more efficiently to Herceptin™ treatment if HER2-Tyr1248 [56] is phosphorylated suggesting that Herceptin™ affects an activated rather than an inactive receptor. However, other sites may influence the response to Herceptin™ [57].

Even if no correlation between HER4 and ER status could be found in our study, the signalling crosstalk of HER receptors with the ERα needs to be functionally explored in more detail. 4ICD has been shown to potentially activate ERα and in turn to stimulate tumour cell proliferation [51]. Accordingly, Tovey and colleagues showed a significantly poorer survival in ER-positive breast cancer patients by detecting nuclear HER4 localisation [58], highlighting the importance of 4ICD/ERα interplay.

Current approaches in developing novel anti-HER receptor targeted drugs that block the entire type 1 RTK family via 'pan-HER' blockade [59] may be counterproductive due to the ambivalent function of HER4. HER4 overexpression or amplification dependent on the prevalent isoform confers a reduced risk [27,54]. Hence development of selective type 1 RTK inhibitors leaving HER4 signalling unaffected may prove more advantageous. On the contrary, antibodies selectively targeting HER4 JM-a, such as the recently introduced monoclonal antibody 1479 [60], could have therapeutic potential, assuming that JM-a-specific antibodies are expected not to cause adverse effects in tissues, such as the heart, that exclusively express ErbB4 JM-b.

## Conclusions

Further studies addressed to elucidate HER4 receptor function in the context of Herceptin™ treatment are necessary to guide the design of highly efficient therapeutic strategies based on HER receptor targeting. Extending the data presented here by stratifying larger patient cohorts characterised by their HER2/HER4 coexpression profile will disclose the impact of HER4 on anti-HER2 targeted Herceptin™ treatment compared with conventional therapy of HER2-positive breast cancer patients. Potentially the evaluation of HER4 expression, by which valuable additional prognostic or even predictive information might possibly be revealed either from immunohistochemistry or FISH even without differentiating between HER4 splice variants, could contribute to therapy decisions and/or optimisation in the future.

## Abbreviations

CI: confidence interval; CT: conventionally treated; DAPI: 4',6-diamidino-2-phenylindole; EGFR: epidermal growth factor receptor; ER: oestrogen receptor; FISH: fluorescence *in situ *hybridisation; H&E: haematoxylin and eosin; HER: human epidermal growth factor receptor; HR: hazard ratio; HT: Herceptin™ treated; IRS: immunoreactive score; OS: overall survival; PR: progesterone receptor; RFS: recurrence free survival; RTK: receptor tyrosine kinase; TMA: tissue microarray.

## Competing interests

The authors declare that they have no competing interests.

## Authors' contributions

AS wrote the manuscript, performed the image analysis and FISH evaluation and analysed the statistical data. MS helped to analyse the statistical data and performed FISH evaluation. SD-D helped to write the manuscript. OO performed the histological analysis and interpreted the data. FH provided material to be analysed and performed primary tissue-based diagnostics. SS performed the histological analysis and interpreted the data. GB is the research group leader, evaluated the data, and finally approved the manuscript to be published. All authors read and approved the final manuscript.
